# Comparison of Texture Features Derived from Static and Respiratory-Gated PET Images in Non-Small Cell Lung Cancer

**DOI:** 10.1371/journal.pone.0115510

**Published:** 2014-12-17

**Authors:** Stephen Yip, Keisha McCall, Michalis Aristophanous, Aileen B. Chen, Hugo J. W. L. Aerts, Ross Berbeco

**Affiliations:** 1 Department of Radiation Oncology, Brigham and Women's Hospital, Dana-Farber Cancer Institute and Harvard Medical School, Boston, Massachusetts, United States of America; 2 Department of Radiology, Dana-Farber Cancer Institute and Harvard Medical School, Boston, Massachusetts, United States of America; 3 Department of Radiation Physics, Division of Radiation Oncology, University of Texas MD Anderson Cancer Center, Houston, Texas, United States of America; 4 Department of Radiology, Brigham and Women's Hospital and Harvard Medical School, Boston, Massachusetts, United States of America; Geisel School of Medicine at Dartmouth College, United States of America

## Abstract

**Background:**

PET-based texture features have been used to quantify tumor heterogeneity due to their predictive power in treatment outcome. We investigated the sensitivity of texture features to tumor motion by comparing static (3D) and respiratory-gated (4D) PET imaging.

**Methods:**

Twenty-six patients (34 lesions) received 3D and 4D [^18^F]FDG-PET scans before the chemo-radiotherapy. The acquired 4D data were retrospectively binned into five breathing phases to create the 4D image sequence. Texture features, including Maximal correlation coefficient (MCC), Long run low gray (LRLG), Coarseness, Contrast, and Busyness, were computed within the physician-defined tumor volume. The relative difference (δ_3D-4D_) in each texture between the 3D- and 4D-PET imaging was calculated. Coefficient of variation (CV) was used to determine the variability in the textures between all 4D-PET phases. Correlations between tumor volume, motion amplitude, and δ_3D-4D_ were also assessed.

**Results:**

4D-PET increased LRLG ( = 1%–2%, p<0.02), Busyness ( = 7%–19%, p<0.01), and decreased MCC ( = 1%–2%, p<7.5×10^−3^), Coarseness ( = 5%–10%, p<0.05) and Contrast ( = 4%–6%, p>0.08) compared to 3D-PET. Nearly negligible variability was found between the 4D phase bins with CV<5% for MCC, LRLG, and Coarseness. For Contrast and Busyness, moderate variability was found with CV = 9% and 10%, respectively. No strong correlation was found between the tumor volume and δ_3D-4D_ for the texture features. Motion amplitude had moderate impact on δ for MCC and Busyness and no impact for LRLG, Coarseness, and Contrast.

**Conclusions:**

Significant differences were found in MCC, LRLG, Coarseness, and Busyness between 3D and 4D PET imaging. The variability between phase bins for MCC, LRLG, and Coarseness was negligible, suggesting that similar quantification can be obtained from all phases. Texture features, blurred out by respiratory motion during 3D-PET acquisition, can be better resolved by 4D-PET imaging. 4D-PET textures may have better prognostic value as they are less susceptible to tumor motion.

## Introduction

Positron emission tomography (PET) with [^18^F]fluorodeoxyglucose (FDG), a surrogate of glucose metabolism, is an essential clinical tool for tumor diagnosis, staging, and monitoring tumor progression [1]–[4]. Accurate quantification of tumor characteristics based on [^18^F]FDG-PET images can provide valuable information for optimizing therapy [5], [6]. Standardized uptake value (SUV) measures such as maximum, peak, mean, and total SUV, are commonly used for quantification of the tumor characteristics [7]–[10]. High baseline SUV uptake has been found to be associated with poor treatment outcome in many tumors, such as esophageal, lung, and head-and-neck cancer [11]–[13].

High intra-tumoral heterogeneity has been shown to relate to poor prognosis and treatment resistance [14], [15]. However, SUV measures fail to adequately capture the spatial heterogeneity of the intra-tumoral uptake distribution [16], [17]. Therefore, texture features, which can be derived from a number of mathematical models of the relationship between multiple voxels and their neighborhood, are proposed to describe tumor heterogeneity [18], [19]. Particularly, pretreatment [^18^F]FDG PET texture features have shown promise for delineating nodal and tumor volumes [20], [21] and assessing therapeutic response [22]–[24]. Studies have suggested that texture features perform better than SUV measures in treatment outcome prediction [22], [24]–[26]. For example, Cook *et al* (2013) compared the predictive power of common SUV measures and four neighborhood gray-tone difference matrix (NGTDM) derived textures in non-small cell lung cancer (NSCLC) patients [27]. They found that NGTDM-derived Coarseness, Contrast, and Busyness were not only better prognostic predictors than the SUV measures, but also better able to differentiate responders from nonresponders.

Despite the clinical potential of texture features, the accurate quantification of texture features may be hindered by respiratory motion in lung cancer patients. Motion induced image blurring in static PET images (3D PET) can lead to reduction in tumor uptake and over estimation of metabolic tumor volume [28]–[30]. 4D PET imaging gates PET image acquisition with respiratory motion in order to improve PET image quality and has been shown to reduce motion blurring in the PET images, providing more accurate quantification of lung tumor activity [28], [31]–[34]. We hypothesize that fine texture features are likely to be blurred during 3D PET acquisition of lung tumors.

With the growing interest of texture features and tumor heterogeneity, the impact of tumor motion on PET-based quantification needs to be studied as it is still yet unknown. In this study, we compared the quantification of texture features between 3D and 4D PET imaging. Although numerous texture features can be found in the literature [22], [35], [36], we focused on five texture features. Particularly, three NGTDM derived Coarseness, Contrast, and Busyness due to their predictive value in lung cancer patients [27]. A gray level co-occurrence matrix (GLCM) derived Maximal Correlation Coefficient (MCC) [37] and gray level run length matrix (GLRLM) derived Long Run Low Gray level emphasis (LRLG) [38] were also computed due to their robustness against variation of reconstruction parameters of PET images [36].

The NGTDM texture features were originally designed to resemble human perception and were first proposed by Amadasun and King (1989) [18]. In a coarse image, the texture is made up by large patterns, such as large area with uniform intensity distribution. Contrast measures the intensity difference between neighboring regions within the tumor. Busyness is a measure of the intensity change between multiple voxels and their surroundings. GLCM-MCC was first introduced by Haralick *et al* in 1973 [37] and is used to measure the statistical relationship between two neighboring voxels. GLRLM-LRLG measures the joint distribution of long runs and low intensity values, where a run is the distance between two consecutive voxels with the same intensity in a specific direction [38].

## Methods

### Patients and imaging

This study was conducted under the Dana-Farber Cancer Institute institutional review board (IRB) approved protocol (protocol #: 06-294) and written consents were obtained from all patients. Twenty-six patients (mean age  = 65±10 yr, 14 males, 12 females) with NSCLC received a treatment planning CT (both 3D and 4D) two weeks before the start of radiotherapy with or without concurrent chemotherapy. 3D [^18^F]FDG-PET/CT, a free breathing chest CT, and a 4D [^18^F]FDG-PET scans were acquired 1–2 weeks prior to the therapy. There were sixteen patients with adenocarcinoma and ten patients with squamous cell carcinoma. The internal tumor volumes (ITV), which encompassed tumor motion, of thirty-four lesions (1–3 malignant tumors/patient) were delineated by an experienced radiation oncologist on a 4D planning CT. 3D PET and 4D PET scans were performed on a Siemens Biograph PET/CT scanner (Siemens AG, Erlangen, Germany). Attenuation correction of 3D PET images was performed using the whole body 3D CT images, while 4D PET images were corrected by the free breathing chest CT images. 3D PET scans were acquired approximately 100 min after injection of 16.7–22mCi of [^18^F]FDG in the patients. For the 3D PET scan, the images were acquired for 3–5 min/bed position in six to seven bed positions. The 3D PET images were reconstructed with ordered-subset expectation-maximization (OSEM) with 4 iterations, 8 subsets, 7 mm full-width-half-maximum (FWHM) post-filtration, and sampled onto a 168×168 grid comprised of 4.06×4.06 mm^2^ pixel. The image acquisition of 4D PET followed immediately after the completion of the 3D PET scan.

4D PET images were acquired at one bed position centered on the tumor and covering part of the lung for 20–30 min, depending on the comfort of the patients. An AZ-733V respiratory gating system (Anzai Medical System, Tokyo, Japan) was employed to monitor patient respiratory motion [39]. The acquired data were retrospectively binned into five phases starting at inhale peak (bin 1) to create the 4D image sequence using the phase-based algorithm provided by the Siemens Biograph PET/CT scanner (Siemens AG, Erlangen, Germany). In particular, the five phase bins, corresponded to the end of inhalation (bin 1), inhalation–to–exhalation (bin 2), mid exhalation (bin 3), end of exhalation (bin4), exhalation–to inhalation (bin 5), respectively. The respiratory gated 4D PET images were reconstructed with OSEM with 2 iterations, 8 subsets, 5 mm FWHM, and sampled onto a 256×256 grid comprised of 2.67×2.67 mm^2^ pixel.

### Texture features

Planning CT was rigidly registered to 3D- and 4D-PET images with normalized mutual information. The transformations were then applied to each ITV. The 3D and 4D PET images were cropped using the registered ITV contour to crop out the tumor region. Number of voxels per tumor region ranged from 85 to 6483 with median number of voxels = 545. Prior to texture feature computation, all PET images (PET(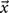)) were preprocessed using the following equation, 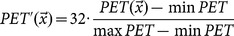
(1)Where minPET and maxPET are the maximum and minimum intensities of PET within the tumor region. The intensity range of the post-processed image (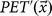) was converted into 32 discrete values as suggested by Orlhac *et al* (2014) [40].

Within the tumor region, the following four neighborhood gray-tone difference matrix (NGTDM) derived texture features were computed to quantify tumor heterogeneity: Coarseness, Contrast, Busyness, and Complexity. These were implemented in MATLAB (The Mathworks Inc. Natrick MA) using the Chang-Gung Image Texture Analysis Toolbox [41], [42]. The mathematical definitions of the NGTDM, GLCM, and GLRLM texture features can be found in Amadasun and King (1989) [18], Haralick *et al* (1973, 1979) [37], [43], and Galloway (1975) [38], respectively.

3D (168×168) and 4D (256×256) PET images were reconstructed to different matrix sizes based on different reconstruction parameters. Additionally, due to the difference in 3D and 4D PET imaging acquisition times, fewer photon counts and higher noise may be found in the 4D PET images. Therefore, all 4D PET images were downsampled to the same grid/resolution of 3D PET images using linear interpolation prior to texture feature computation to reduce noise.

### Data analysis

The relative difference (δ_3D-4D_) in texture features between 3D and 4D PET were calculated: 
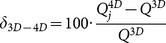
(2)Where 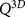 is the quantification (i.e. texture features measures) based on 3D PET, 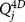 is the quantification based on bin *j* of the 4D PET images. Wilcoxon signed-rank test (p<0.05) was performed on pairs to determine if 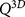 and 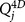 were significantly different. We calculated an avid tumor volume (ATV) as thresholded PET images with SUV over 40% maximum SUV within the ITV [29]. We investigated the influence of ATV and ITV on δ_3D-4D_ using Spearman's correlation coefficient (R) with significant value of p = 0.05.

Kruskal-Wallis test was used to assess if one phase was significantly different from the other phases (p<0.05). The variability in the texture features measures between all five phase bins was assessed using the coefficient of variation (CV). 
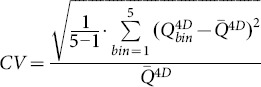
(3)




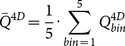
(4)To estimate the extent of motion, the centers of mass (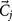) of the PET avid region (ATV) on all five 4D PET bins were recorded. The amplitude of the tumor motion was estimated using the maximum difference in 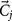 between the five bins [28], [29]

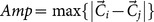
(5)Where *i* and *j* range from 1 to 5.

To study the impact of tumor motion, we calculated the Spearman's correlation coefficient for Amplitude:ATV ratio and δ_3D-4D_ with significant value p = 0.05. Amplitude:ATV ratio is a measure of motion amplitude relative to the tumor volume. Large Amplitude:ATV ratio indicates large tumor movement relative to the tumor size.

Furthermore, textures may be affected by motion differently according to the tumor histology. Therefore, we investigated if δ_3D-4D_ were significantly different between adenocarcinomas (21 lesions) and squamous cell carcinomas (13 lesions) using Mann-Whitney U-test with p<0.05.

## Results

**Figure 1:**
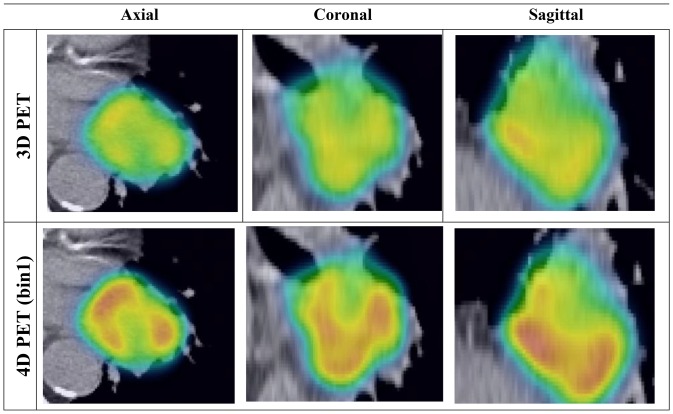
3D (top row) and 4D (bottom row) PET images overlaid onto the 3D CT. All images are displayed in the same intensity window with SUV between 1 and 15.

**Figure 2:**
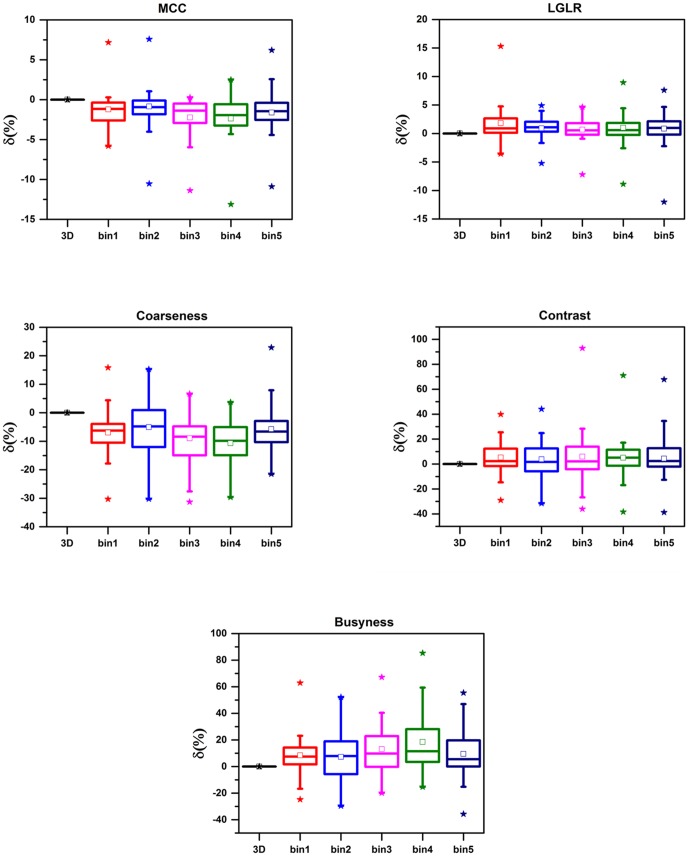
Distribution of the difference between 3D and 4D PET (δ_3D-4D_) in the texture features across 34 lesions. The top vertical line of a boxplot represents 75^th^—95^th^ percentiles of the data. The bottom vertical line is the 5^th^—25^th^ percentiles. Interquartile range (IQR) of the data is indicated by the width of the boxplot. Asterisks indicate the maximum and minimum differences. Median and mean differences are indicated by bar and square inside the box plots, respectively. MCC = Maximal correlation coefficient. LRLG = Long run low gray-level emphasis. The first boxplot represents the comparisons of 3D and 3D PET textures (δ_3D-3D_). δ_3D-3D_ is therefore zero by definition as shown in the first “boxplot” for each texture.

4D PET images appeared to have higher uptake and less blurring than the corresponding 3D PET images (Fig. 1). The differences between 3D and 4D PET were found to be significant (p<<0.01) for Busyness, MCC, and LRLG as shown in Table 1. Significant difference for Coarseness was found in all bins (p<<0.01) except in bin 2 (p = 0.59) (Table 1). The Coarseness determined on the 3D PET images was about 10% higher than the 4D PET. 4D PET images were found to have as much as a 19% increase in Busyness, compared to the corresponding 3D PET images (Table 1, Fig. 2). MCC was found to be 2% higher in 3D PET than 4D PET, while 2% higher LRLG was found in 4D PET when comparing to 3D PET. However, Contrast on 3D images was only about 5% lower when compared to 4D PET and δ_3D-4D_ was not significant (p>0.08) (Table 1, Fig. 2).

**Table 1:** The mean difference (δ_3D-4D_) between 3D and 4D PET images in texture features.

	Bin-1	Bin-2	Bin-3	Bin-4	Bin-5
MCC	−1±2%	−1±3%	−3±2%	−3±3%	−2±3%
	(−6%–7%)	(−11%–8%)	(−11%–0%)	(−13%–2%)	(−11%–6%)
	p = 2.0×10^−4^	p = 7.5×10^−3^	p = 6.2×10^−7^	p = 3.8×10^−6^	p = 1.4×10^−4^
LRLG	2±3%	1±2%	1±2%	1±3%	1±3%
	(−4%–15%)	(−5%–5%)	(−7%–5%)	(−9%–9%)	(−12%–8%)
	p = 1.5×10^−3^	p = 2.4×10^−3^	p = 0.02	p = 9.6×10^−3^	p = 8.3×10^−3^
Coarseness	−7±8%	−5±10%	−9±9%	−11±8%	−6±10%
	(−30%–16%)	(−30%–15%)	(−31%–7%)	(−30%–4%)	(−21%–23%)
	p = 4.1×10^−4^	p = 0.05	p = 1.1×10^−4^	p = 1.0×10^−4^	p = 2.3×10^−3^
Contrast	5±14%	4±15%	6±22%	5±18%	4±19%
	(−29%–40%)	(−32%–44%)	(−36%–93%)	(−38%–71%)	(−39%–68%)
	p = 0.08	p = 0.72	p = 0.54	p = 0.12	p = 0.55
Busyness	8±16%	7±18%	13±18%	19±24%	9±18%
	(−25%–63%)	(−30%–52%)	(−20%–67%)	(−15%–85%)	(−36%–55%)
	p = 1.4×10^−3^	p = 0.01	p = 1.3×10^−4^	p = 3.0×10^−5^	p = 7.3×10^−4^

The ranges of δ_3D-4D_ and the p-values for Wilcoson signed-rank test are also shown. MCC = maximal correlation coefficient. LRLG = Long run low gray-level emphasis

None of the phases were significantly different from the other for any texture features (p>0.90, Kruskal-Wallis test). Negligible to moderate variability in the texture features was found between the five phase bins (Fig. 2). CV was only 1% for MCC and LRLG, 5% for Coarseness, 9% and 10% for Contrast and Busyness, respectively. The avid tumor volume (ATV) was poorly correlated to δ_3D-4D_ for all texture features (R = −0.24–0.38, p = 0.03–0.07). The correlation between internal tumor volumes (ITV) and δ_3D-4D_ were also found to be poor for all textures (R = −0.31–0.30, p>0.02), except LGLR. Although δ_3D-4D_ for LGLR was moderately influenced by ITV (R = −0.62–−0.31, p = 8.3×10^−5^–0.08), the average δ_3D-4D_<2%.

Average motion amplitude was found to be 4.4±4.6 mm (0.6–20.5 mm). As shown in Table 2, moderate to substantial correlation was found between Amplitude:ATV (mm^−2^) and δ_3D-4D_ for Busyness (R = 0.38–0.54) and MCC (R = −0.70–−0.41) in bin 3–5, whereas poor correlation was found in bin 1–2 with R = −0.03–0.12. The correlations were also poor for Coarseness (R = −0.32–0.18), Contrast (R = −0.35–−0.10), and LRLG (R = 0.08–0.34) (Table 2). Moreover, δ_3D-4D_ were not significantly different between the histologies, adenocarcinomas and squamous cell carcinomas, with p>0.26 (Table 3).

**Table 2:** Spearman correlation coefficient of Amplitude:ATV (mm^−2^) and δ_3D-4D_ and its p-value.

	Bin-1	Bin-2	Bin-3	Bin-4	Bin-5
MCC	−0.07	0.12	*−0.70*	*−0.62*	*−0.41*
	p = 0.71	p = 0.51	*p = 4.3×10^−6^*	*p = 1.1×10^−4^*	*p = 0.02*
LRLG	*0.34*	0.27	0.08	0.24	0.19
	*p = 0.05*	p = 0.11	p = 0.64	p = 0.16	p = 0.28
Coarseness	0.05	0.18	−0.32	−0.23	0.06
	p = 0.78	p = 0.31	p = 0.07	p = 0.19	p = 0.74
Contrast	−0.14	−0.20	−0.10	−0.23	−0.35
	p = 0.44	p = 0.26	p = 0.59	p = 0.18	p = 0.04
Busyness	0.00	−0.03	*0.43*	*0.54*	*0.38*
	p = 0.99	p = 0.88	*p = 0.01*	*p = 9.3×10^−4^*	*p = 0.03*

MCC = Maximal correlation coefficient. LRLG = Long run low gray-level emphasis.

**Table 3:** p-values for the comparison of δ_3D-4D_ between adenocarcinoma and squamous cell carcinoma using Mann-Whitney U-test.

	Bin-1	Bin-2	Bin-3	Bin-4	Bin-5
MCC	p = 0.48	p = 0.77	p = 0.53	p = 0.90	p = 0.84
LRLG	p = 0.77	p = 0.26	p = 0.48	p = 0.30	p = 0.49
Coarseness	p = 0.87	p = 0.61	p = 0.79	p = 0.55	p = 0.55
Contrast	p = 0.46	p = 0.68	p = 1.00	p = 0.66	p = 0.45
Busyness	p = 0.59	p = 0.80	p = 0.93	p = 0.86	p = 0.78

## Discussion

In this study, we investigated the sensitivity of prognostic PET texture features to respiratory motion. Our results suggest that texture measures are sensitive to tumor motion. Substantial differences between 3D and 4D (δ_3D-4D_ >10%) were found in Coarseness and Busyness. Therefore, the temporal resolution offered by 4D PET imaging may lead to more accurate quantification of image features.

Coarseness, Contrast, and Busyness considered in this study were originally designed to resemble human perception and were first proposed by Amadasun and King (1989) [18]. Cook *et al* (2012) [27] have shown that these three texture features are clinically relevant to lung cancer due to their predictive value for patient outcome. In a coarse image, the texture is made up by large patterns, such as large area with uniform intensity distribution. As breathing motion blurs the fine textures in the images, the 3D PET images appear to be more uniform (Fig. 1) and therefore have more Coarseness than 4D PET images. The sensitivity of Contrast was found to be insignificant to motion induced blurring. The intensity difference between neighboring regions within the tumor was observed to be more pronounced in 4D PET image (Fig. 1), leading to slightly higher (δ_3D-4D_∼5%) Contrast in 4D PET than 3D PET images. Busyness is a measure of the intensity change between single voxels and their surroundings. Busyness computed with 4D PET images was found to be as much as 20% higher than the 3D PET images. Since δ_3D-4D_ tended to be higher at large Amplitude:ATV, the quantification of Busyness is especially sensitive to large relative tumor amplitude. However, 3D PET imaging was employed in the study of Cook *et al* (2012). Our results suggest that the quantification and prognostic value of busyness can be adversely affected by tumor motion.

GLCM-MCC and GLRLM-LRLG were included in the 3D vs 4D PET imaging comparison as they are insensitive to reconstruction parameters of PET images [36]. Tumor motion blurring in 3D PET image can reduce intensity difference between neighboring voxels. Therefore, neighboring voxels are better correlated in 3D PET than 4D PET, leading to significant 2% higher MCC in 3D PET images. LRLG measures the joint probability of long runs and low gray values. As observed in Fig. 1, low intensity voxels are more localized (less distance apart) in the motion blurred 3D PET than in the 4D PET images. Therefore, LRLG was higher in 4D PET than 3D PET.

In this study, the 4D PET images were binned into five phases. The activity uptake of each bin was slightly different as in Huang and Wang (2013) [30]. The bin with the highest SUV_max_ is often chosen to be the “best” bin for 4D PET image [29], [44], [45]. However, we found that the variability between phase bins for MCC, LRLG, and Coarseness were negligible (CV<5%), suggesting that similar quantification can be obtained from all phases. The small variability may be due to the small tumor amplitude (4.4±4.6 mm) in our dataset. On the other hand, the phase bin variability was found to be moderate for Contrast and Busyness (CV∼10%). The values of Contrast and Busyness may depend on the choice of phase-bin. MCC, LRLG, and Coarseness are independent of the choice of phase-bin, and therefore should be recommended for quantification of tumor characteristics in 4D PET imaging.

Apart from the texture features, studies often investigate the effect of respiratory motion on the quantification of various SUV measures, especially the maximum SUV [28], [29], [33]. The SUV_max_ was found to increase with 4D PET imaging from 25% to 80% in these studies. The motion induced artifacts not only lower maximum tumor uptake on the 3D PET images, but may also lead to misclassification of lesions. For example, García Vicente *et al* (2010) compared the SUV_max_ determined on 3D and 4D PET images for 42 lesions in lung cancer patients [33]. Tumor with SUV_max_ over 2.5 was considered malignant in their study. As a result, 40% (17/42) of the lesions needed to be changed from benign to malignant. To this end, although the results are not shown, we also compared the differences in four SUV measures (SUV_max_, SUV_peak_, SUV_mean_, and SUV_total_). 4D PET imaging increased the measurements of SUV_max_ and SUV_peak_ by about 30% and 25%, respectively, while increased for SUV_mean_ and SUV_total_ were only about 5%. Our results in SUV_max_ are comparable to the previous studies [28], [29], [33].

However, there is one limitation of our textures and SUV comparison as it has been shown that malignant tumor tissue can continuously increase the uptake of [^18^F]FDG even 2 hours after injection [46]–[48]. While the 3D PET imaging was acquired about 100 min after the [^18^F]FDG-PET injection, 4D PET imaging was acquired between 118–135 min after injection. Therefore, the increase in [^18^F]FDG-PET seen in our study may not be due solely to respiratory motion. Dong *et al* (2013) found a significant correlation between SUV_max_ and textures (entropy and energy) derived from PET intensity histograms in patients with esophageal cancer [49]. SUV_max_ was also found to be highly correlated to entropy and energy in a study conducted by Orlhac *et al* (2014) [40] using patients with metastatic colorectal, lung, and breast cancer. These two studies may therefore suggest that the histogram derived textures are likely to be affected by the delayed imaging. However, none of the textures that were used in our study has been found to be highly correlated with SUV_max_ [40]. This may be due to the fact that the textures we used are based on the spatial relationship between neighborhoods of voxels, and are not directly dependent on the intensity value of single or multiple voxels within the tumors. However, further study is needed to better understand the impact of delayed imaging on texture quantification.

All the PET images in our study underwent attenuation correction using the free breathing CT images. The blurred anatomical mismatched of the PET/CT scans due to respiratory motion may affect the quality of the attenuation corrected 4D PET images, and subsequently the quantification of texture features [29], [50], [51]. Moreover, due to the difference in 3D and 4D PET imaging acquisition times, fewer photon counts and higher noise may be found in 4D PET images, which may subsequently affect the accuracy of texture feature definition. To mitigate the effect of noise, all 4D PET images have a minimum acquisition time of 20 min. These potential effects will be explored further in a future study.

## Conclusions

Texture features, representing tumor heterogeneity, are blurred out by respiratory motion during 3D PET acquisition. 4D PET imaging reduces motion blurring, enabling PET-based features to be better resolved. Significant differences were found in MCC, LRLG, Coarseness, and Busyness between 3D and 4D PET imaging. When measuring tumor heterogeneity characteristics with PET imaging, reduced motion blurring by 4D PET acquisition enables significantly better spatial resolution of texture features. 3D PET textures may lead to inaccurate prediction of treatment outcome, hindering optimal lung cancer patient management. 4D PET textures may have better prognostic value as they are less susceptible to tumor motion.
